# *Babesia pisicii* n. sp. and *Babesia canis* Infect European Wild Cats, *Felis silvestris*, in Romania

**DOI:** 10.3390/microorganisms9071474

**Published:** 2021-07-09

**Authors:** Luciana Cătălina Panait, Kristýna Hrazdilová, Angela Monica Ionică, Georgiana Deak, Gabriel Bogdan Chişamera, Costică Adam, Călin Mircea Gherman, Andrei Daniel Mihalca

**Affiliations:** 1 Department of Parasitology and Parasitic Diseases, Faculty of Veterinary Medicine, University of Agricultural Sciences and Veterinary Medicine of Cluj-Napoca, 400372 Cluj-Napoca, Romania; georgiana.deak@usamvcluj.ro (G.D.); calin.gherman@usamvcluj.ro (C.M.G.); amihalca@usamvcluj.ro (A.D.M.); 2 CEITEC VETUNI, University of Veterinary Sciences Brno, 61242 Brno, Czech Republic; kristyna@hrazdilova.cz; 3 Biomedical Center, Faculty of Medicine in Pilsen, Charles University, 32300 Plzeň, Czech Republic; 4 Molecular Biology and Veterinary Parasitology Unit (CDS-9), “Regele Mihai I al României” Life Science Institute, University of Agricultural Sciences and Veterinary Medicine of Cluj-Napoca, 400372 Cluj-Napoca, Romania; ionica.angela@usamvcluj.ro; 5 “Grigore Antipa” National Museum of Natural History, 011341 Bucharest, Romania; gabriel.chisamera@gmail.com (G.B.C.); cadam@antipa.ro (C.A.)

**Keywords:** *Babesia pisicii* n. sp., European wild felids, piroplasmids, 18S rDNA, mitochondrial genes

## Abstract

Haemoparasites of the genus *Babesia* infect a wide range of domestic and wild animals. Feline babesiosis is considered endemic in South Africa, while data on *Babesia* spp. infection in felids in Europe is scarce. Using samples from 51 wild felids, 44 *Felis silvestris* and 7 *Lynx lynx*, the study aimed to determine the presence and genetic diversity of *Babesia* spp. in wild felids in Romania by analyzing the 18S rDNA and two mitochondrial markers, cytochrome *b* (Cytb) and cytochrome *c* oxidase subunit I (COI) genes. By 18S rDNA analyses, *Babesia* spp. DNA was detected in 20 European wild felids. All sequences showed 100% similarity to *B. canis* by BLAST analysis. Conversely, Cytb and COI analyses revealed the presence of two *Babesia* spp., *B. pisicii* n. sp., which we herein describe, and *B. canis*. The pairwise comparison of both mitochondrial genes of *B. pisicii* n. sp. showed a genetic distance of at least 10.3% from the most closely related species, *B. rossi*. Phylogenetic analyses of Cytb and COI genes revealed that *B. pisicii* n. sp. is related to the so-called “large” canid-associated *Babesia* species forming a separate subclade in a sister position to *B. rossi*.

## 1. Introduction

The genus *Babesia* is composed of apicomplexan tick-transmitted haemoparasites with a remarkable economic, medical, and veterinary impact on domestic and wild animals [1,2,3]. Moreover, *Babesia* species are gaining increased interest as potential etiological agents of zoonotic diseases [4,5]. Since the first description of the microorganism in erythrocytes of Romanian cattle by Victor Babeş, at the end of the 19th century, more than 100 new species have been described [6,7]. The growing number of available mitochondrial sequences suggests that species diversity of piroplasmids in European wildlife is highly underestimated [8,9]. Robust species differentiation and understanding of their hosts spectrum are necessary for the identification of the parasite in endangered wild species, and for proper diagnostic of the clinical cases in domestic animals.

Feline babesiosis is considered endemic in South Africa, although *Babesia* spp. have been sporadically reported from various countries from Europe, Asia, or America [10]. In the previous century, several *Babesia* spp. have been described based only on morphological characteristics or host specificity, including *B. felis, B. cati, B. herpailuri*, and *B. pantherae* [11,12,13,14]. However, only *B. felis* has been molecularly characterized thereafter [15]. Based on molecular data, *B. leo*, *B. lengau*, and *Babesia* species cat Western Cape were also documented in South Africa [15,16,17]. In Asia, *B. canis presentii* and *B. hongkongensis* have been reported and molecularly characterized in domestic cats [18,19]. Additionally, *B. microti* as well as dog-related species such as *B. canis*, *B. gibsoni*, and *B. vogeli* have been identified in felids based on molecular data [20,21,22,23]. The vast majority of deposited sequences are represented by small ribosomal RNA subunit gene (18S rDNA) sequences, while few internal transcribed spacer, 5S rRNA, 28S rRNA, or beta tubulin-like gene sequences are available. However, only one mitochondrial sequence is accessible for comparison (cytochrome *b* (Cytb) of *B. hongkongensis*, accession number JQ867357), which may limit phylogenetic analyses of feline specific *Babesia* spp.

In Europe, data on *Babesia* spp. infections in felids are sporadic and inconsistent. In European wild felids, *Babesia* was reported in one individual from Bosnia and Herzegovina and it was molecularly characterized as identical to *Babesia* sp. previously found in badgers [24]. The presence of intraerythrocytic merozoites compatible with small *Babesia*/*Cytauxzoon* spp. was documented in a wild cat in northern Greece; however, the results are not supported by molecular data [25]. In domestic cats, molecular findings of *B. canis*, *B. vogeli*, or *B. microti* were reported in Spain, Portugal, Poland, and Italy [20,22,26,27]. This is in high contrast with numerous reports of *Babesia* infection in domestic dogs in Europe during the last decades, with prevalence rates ranging from 0.1 to 88.0% [28].

The present study was driven by the lack of relevant data on *Babesia* in European wild felids and by our findings of *B. canis* in felids in the course of other studies on feline piroplasms [9]. Therefore, we aimed to understand the diversity of feline *Babesia* in wild felids in Romania using the 18S rDNA and subsequent molecular characterization by analyzing two mitochondrial markers, Cytb and cytochrome *c* oxidase subunit I (COI) genes.

## 2. Materials and Methods

### 2.1. Samples

**Figure 1:**
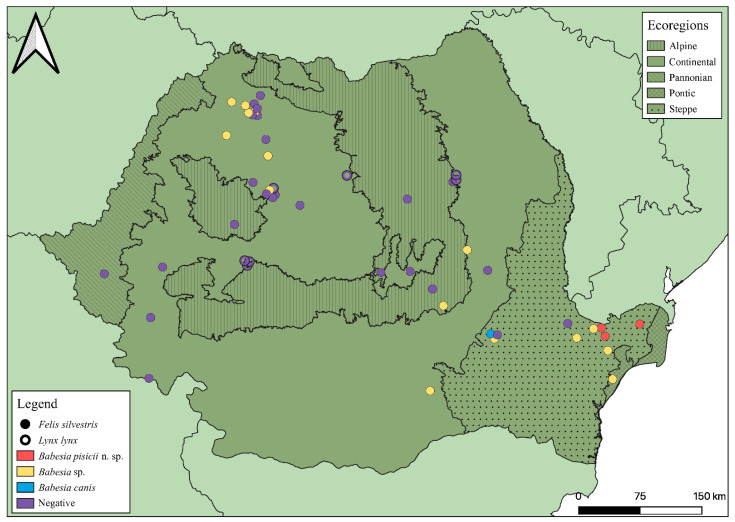
Geographical distribution of the samples included in the study and their positivity to *Babesia* sp. (samples that yielded a positive result in the assay targeting 18S rDNA fragment of 376 bp), *B. canis*, and *B. pisicii* n. sp. (confirmed by mitochondrial genes analysis).

Between February 2011 and February 2020, 51 wild felids carcasses (44 *Felis silvestris* and 7 *Lynx lynx*) were examined at the Department of Parasitology and Parasitic Diseases of the University of Agricultural Sciences and Veterinary Medicine of Cluj-Napoca, Romania. The animals were found as road kill or died of natural causes. During necropsy, blood, spleen, liver or heart samples were collected and stored at −20 °C, until further processing. Samples from 42 animals were included in previous studies [9,29], focusing on *Cytauxzoon* spp. detection and characterization. The wild felid species identification was carried out based on different morphological and pelage characters [30]. If available, data on origin was recorded for each animal (Figure 1). The study area was divided into five ecoregions: continental, steppe, alpine, Pannonian, and Pontic, as previously described [31].

### 2.2. DNA Isolation, PCR Amplification, and Phylogenetic Analyses

Genomic DNA was extracted from 200 μL of whole blood or 20 mg of tissue using Isolate II Genomic DNA Kit (Bioline, London, UK), according to manufacturer’s instruction.

A partial fragment of the 18S rDNA of *B. canis* was amplified using a species-specific nested PCR protocol. Subsequently, all the samples that yielded a positive result were screened by PCR assays targeting a longer fragment of the 18S rDNA, mitochondrial Cytb, and COI genes. Primers, annealing temperatures, and expected length of amplicons are listed in Table 1. Amplification of the first-round reactions were performed in 15 μL reaction mixture, containing 400 nM of each primer, 7.5 μL of 2× PCRBIO Taq Mix Red (PCR Biosystems, London, UK), and 1 μL of template DNA. The second-round reactions were carried out in a total volume of 25 μL, consisting of 400 nM of each primer, 12.5 μL of 2× PCRBIO Taq Mix Red (PCR Biosystems, London, UK), and 1 μL of primary product. PCR products were visualized by electrophoresis on 1.5% agarose gels stained with ECO Safe Nucleic Acid Staining Solution (PacificImage Electronics, New Taipei City, Taiwan).

PCR products of expected size were excised from gels, purified using Gel/PCR DNA Fragment Extraction Kit (Geneaid Biotech, New Taipei City, Taiwan), and sequenced bi-directionally (Macrogen, Amsterdam, the Netherlands) using the amplification primers. Sequence chromatograms were edited using Geneious 9.1.2 [32] and compared with representative sequences available in the GenBank database by NCBI Basic Local Alignment Search Tool (BLAST) analysis. Alignments of 18S rDNA sequences were generated using ClustalW algorithm.

The phylogenetic trees of Cytb and COI genes were based on sequences acquired in this study and all available sequences of corresponding genes from *Babesia* sensu stricto species [35] from GenBank longer than 300 nt (sequences containing premature STOP codon in the open reading frame were excluded from the analyses). Four Cytb and three COI gene sequences of *Theileria* spp. from GenBank were used as an outgroup. The alignments on nucleotide level were guided by amino acid translation (TransAlign, Geneious 9.1.2), restricted to protein coding regions only. The resulting alignments were built from 80 sequences (1440 nt) for COI and 60 sequences (1098 nt) for Cytb. For chosen closest species, alignments were also prepared as described above and their p-distances were computed by Geneious 9.1.2.

All phylogenetic trees were inferred by maximum likelihood method using IQ-TREE v. 1.6.5 [36]. The best-fit evolution models (K3Pu + F + G4 for Cytb gene and GTR + F + I + G4 for COI gene) were chosen based on the Bayesian information criterion (BIC) computed by ModelFinder [37]. Branch supports were assessed by the ultrafast bootstrap (UFBoot) approximation [38] and by SH-like approximate likelihood ratio test (SH-aLRT) [39]. Trees were visualized and edited in FigTree v1.4.4 and Inkscape 0.94.

### 2.3. Sensitivity of the Assay Targeting 18S rDNA Fragment of 376 bp

The sensitivity of detection of the PCR protocol targeting the 376 bp region of the *B. canis* 18S rDNA was assessed as described elsewhere [34]. The concentration and purity of the linearized plasmids was evaluated in triplicates by NanoDrop ND-1000 spectrophotometer analyzer (NanoDrop Technologies, Inc., Wilmington, DE, USA). The number of molecules were calculated using the formula:

(1) $$number\;of\;DNA\;copies=\left(dsDNA\times N_{A}\right)/\left(lenght\times 662\right),$$

where *dsDNA* is the amount of DNA [g/μL], *N_A_* is the Avogadro’s number $\left[6.022\times 10^{23}{\;\mathrm{mol}}^{-1}\right]$, *length* is the length of target sequence including vector [bp], and 662 is the average molecular weight of a base pair [g/mol]. Ten-folds dilution series were prepared by combining linearized pGEM^®^-T Easy plasmid containing insert with DNA isolated from *Babesia* spp. negative wild cats. The final aliquots, with a concentration of 1 to 10^5^ copies/μL, were used as template in the nested PCR protocol described above. From both PCR rounds, 10 μL of the final product was visualized on 1.5% agarose gels stained by ECO Safe Nucleic Acid Staining Solution, purified and submitted for sequencing on both strands (Macrogen, Amsterdam, The Netherlands).

## 3. Results

### 3.1. 18S rDNA Sequence Analyses

*Babesia* spp. infection was detected by amplification and sequencing of the 376 bp fragment of the 18S rDNA in 20 wild felids (39.2%; 95% CI: 27.0–52.9). All obtained sequences were identical to each other, and the BLAST analysis showed 100% similarity to *B. canis* isolated from dogs from Lithuania, Iran, or Bosnia and Herzegovina, or red foxes from Poland, etc. (GenBank accession numbers: MN078319-MN078323, MN173223, MN134074, MK107806). All the positive samples originated from European wild cats, while none of seven Eurasian lynxes were found positive. The geographical origin of the samples included in the study and their positivity are shown in Figure 1.

From the 20 positive *F. silvestris* samples, 14 (27.5%; 95% CI: 17.1–41.0) yielded an amplicon in the assay targeting the 1670 bp fragment of the 18S rDNA gene. However, the presence of *B. canis* was confirmed only in two samples by direct sequencing (100% identity to *B. canis* from Romania and Estonia; GenBank accession numbers: KX712122, HQ662634, KT008057). The other amplicons represent members of the genus *Cytauxzoon* and *Hepatozoon*, co-amplified by the assay.

### 3.2. Evaluation of Assay Sensitivity Targeting the 376 bp Fragment of the 18S rDNA

The sensitivity of the nested PCR assay targeting the 376 bp fragment of the 18S rDNA was established to a single molecule in a reaction after the second round of PCR. In the first PCR round, the detection limit was 10^2^ copies of template DNA (Supplementary file: Figure S1). Direct sequencing confirmed the presence of *B. canis* 18S rDNA in all PCR products that yielded visible bands from both PCR rounds.

### 3.3. Mitochondrial Genes Analyses

To assess the genetic variability of *Babesia* spp. present in wild felids, sequence analyses of two mitochondrial genes (Cytb and COI) were performed. Amplification of the Cytb gene fragment was successful in four out of the 20 18S rDNA positive samples (7.8%; CI: 3.1–18.5). Direct sequencing yielded high-quality *Babesia* spp. consensus sequences of 556–582 nt for all these four samples. The sequence from sample 3569 showed 99.8% identity to *B. canis* from USA (accession number KC207822) by BLAST analysis. The closest relative of the remaining three sequences (100% identical to each other) was *B. rossi* with 87.2–89.7% identity (accession number KC207823). All these three sequences originated from wild cats from the steppe ecoregion (Figure 1).

The mitochondrial COI marker was amplified from the same four samples. The sequence from sample 3569 showed 99.7% identity to the aforementioned *B. canis* isolate from USA (accession number KC207822). The other three sequences (99.9–100% identical to each other) also showed an identity of 84.3–89.7% to the same *B. rossi* (accession number KC207823).

The pairwise comparison of the mitochondrial genes of this new *Babesia* genotype showed a genetic distance of at least 10.3% in both Cytb and COI genes from the most closely related species, *B. rossi*. Even higher nucleotide sequence distances were obtained between the new genotype and *B. canis* or *B. vogeli* (Table 2, sequence distances on amino acid level are available in the supplementary file: Table S1).

**Figure 2:**
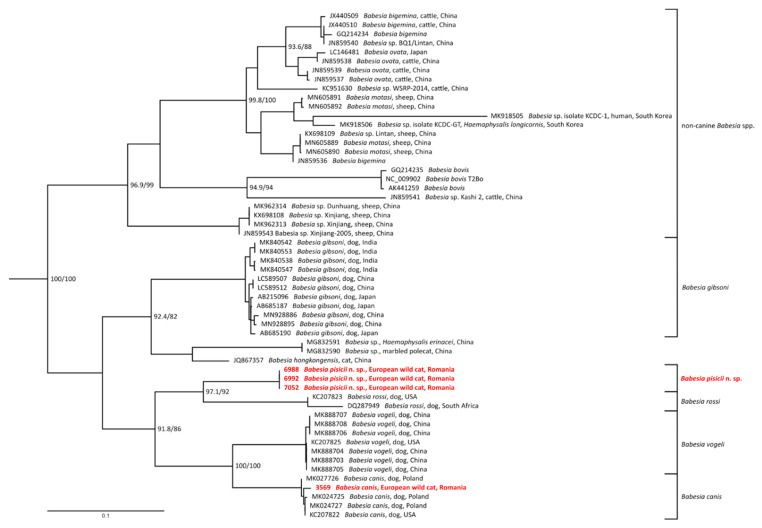
Phylogenetic tree assessed by maximum likelihood method based on Cytb sequences of *Babesia* sensu stricto species (clade X. sensu Jalovecká et al. [35]) and sequences obtained during the current study (highlighted in red); bootstrap values (SH-aLRT/UFB) above the threshold 80/95 are displayed. The scale bar indicates the number of nucleotide substitutions per site.

**Figure 3:**
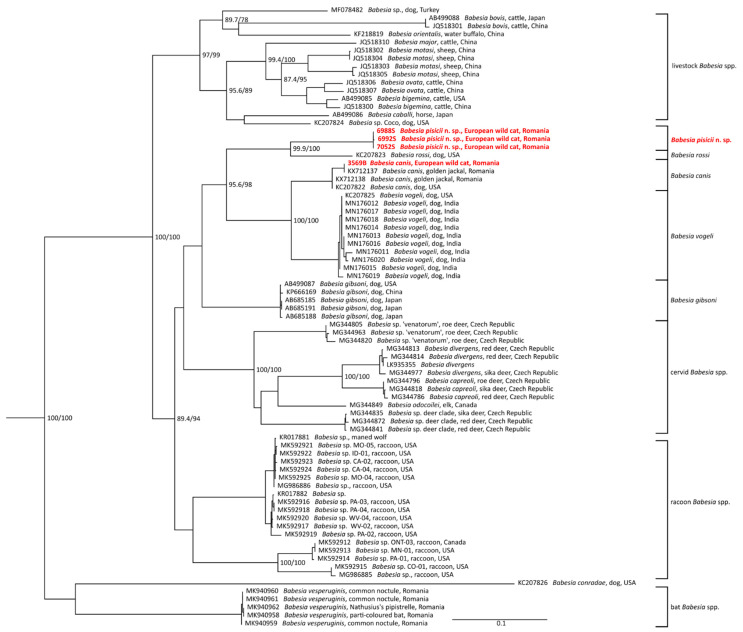
Phylogenetic tree assessed by maximum likelihood method based on COI sequences of *Babesia* sensu stricto species (clade X. sensu Jalovecká et al. [35]) and sequences obtained during the current study (highlighted in red); bootstrap values (SH-aLRT/UFB) above the threshold 80/95 are displayed. The scale bar indicates the number of nucleotide substitutions per site.

Phylogenetic analyses of both mitochondrial genes were based on GenBank available sequences of *Babesia* sensu stricto species (clade X sensu Jalovecká et al. [35]) and sequences obtained during the current study. All the new *Babesia* genotype sequences formed a distinct and highly supported subclade in both Cytb and COI phylogenies, in a sister position to *B. rossi* (Figure 2 and Figure 3). Furthermore, this subclade is placed more distantly from the sequences representing the *B. vogeli* and *B. canis* clade. The *B. canis* sequences from this study, clustered together with other published Cytb and COI sequences of *B. canis*.

All the unique sequences obtained in this study were deposited in GenBank database under the accession numbers MW939359 (18S rDNA), MW938761 (Cytb gene), MW938763 (COI gene) for *B. canis* and MW939360 (18S rDNA), MW938762 (Cytb gene), MW938764-MW938765 (COI gene) for the new *Babesia* genotype.

Based on these data, we herein describe this genotype as a new species of *Babesia*.

### 3.4. Taxonomic Summary and Species Description

Order Piroplasmida Poche, 1913.

Family Babesiidae du Toit, 1918.

Genus *Babesia* Starcovici, 1893.

*Babesia pisicii* n. sp. Panait, Hrazdilová, and Mihalca.

***Diagnosis:*** the organism is a species of piroplasmid protist of the genus *Babesia*, distinctive from congeners from other carnivores based on DNA sequences and forming a separate clade sister to *B. rossi*.

***Type-host:*** *Felis silvestris* Schreber, 1777 (Carnivora: Felidae).

***Type-locality:*** Mila 23, Tulcea (45.22° N, 29.24° E).

***Type-material:*** tissue extract and the total DNA isolated are deposited at the “Grigore Antipa” Natural History Museum, Bucharest, Romania under collection numbers BAB 001 (the tissue) and BAB 002 (the DNA). In correspondence with the ICZN code (Arts 72.5.4, 73.3) [40], the material deposited is considered a hapantotype by its character.

***DNA sequences:*** DNA sequences amplified from the type material are deposited in GenBank under the accession numbers: MW939360 (18S rDNA), MW938762 (Cytb gene), MW938764 (COI gene).

***Other localities:* Romania**: Cataloi, Tulcea (45.10° N, 28.72° E), Somova, Tulcea (45.19° N, 28.67° E).

***Prevalence:*** 3/51 (5.9%)

***Etymology:*** the specific epithet *pisicii* derives from the Romanian term used for cats. The name is given as a genitive noun, according to the ICZN rules and recommendations.

## 4. Discussion

Historically, *Babesia* spp. differentiation was based on the assumed host specificity and phenotypic characteristics, such as the size of intraerythrocytic stages and the number of merozoites observed during microscopic visualization of the blood smears [2]. Currently, the development of molecular techniques and the availability of extensive molecular data have questioned host specificity and allowed species identification [34,41,42].

In Europe, intraerythrocytic parasites of the genus *Babesia* were molecularly identified in a wide range of mammalian hosts, including bovines [43,44], small ruminants [45,46], different deer species [8], equines [47], swine [48], laboratory rodents [49], hares [50], moles [51], bats [52], and various carnivores such as dogs [34,53], wolves [54], jackals [55], foxes [56], cats [26,27], and mustelids [57].

The frequent use of universal primers detecting the 18S rDNA of a wide range of apicomplexan parasites (*Babesia–Theileria–Hepatozoon–Cytauxzoon*) [24,58,59,60] may often lead to amplification of other parasites than *Babesia* spp. in samples co-infected with other blood apicomplexans, such as *Cytauxzoon* spp. [9,61] and *Hepatozoon* spp. [24,62]. As a consequence, there is a single report of *Babesia* sp. in a wild cat [24] in Europe.

Although *B. canis* is typically considered a canid-associated species, during the last decades, its presence was reported also in non-canid hosts such as bats [63], horses [64], and domestic cats [20,26]. However, all of these reports are based on 18S rDNA detection, which has limitations in distinguishing very closely related *Babesia* species due to the insufficient sequence variation [2,34,65]. Moreover, the DNA of *B. canis* was recently demonstrated to be detectable in mice experimentally fed with naturally infected *Dermacentor reticulatus* ticks [66]. Thus, the detection of *B. canis* using 18S rDNA assays in felids could be related to non-specific detection of closely related species or to a possible ingestion of *B. canis* sensu stricto from prays or their ticks.

Piroplasmid species delineation based on mitochondrial sequences has been recently demonstrated and applied for description of closely related species [9]. Even if sequences from 39.2% of the tested samples showed 100% similarity to *B. canis* after the short fragment of the 18S rDNA analysis, the results obtained in mitochondrial markers assays confirmed the presence of *B. canis* in only one sample, while *B. pisicii* n. sp. was identified in three individuals. Therefore, an accurate specific identification of piroplasms should be followed by species confirmation by mitochondrial markers. However, protocols used to amplify mitochondrial genes have 100 to 1000 times lower sensitivities than the specific assay targeting the 376 bp fragment of the *B. canis* 18S rDNA, as demonstrated by Hrazdilová et al. [34].

In this study, fresh blood was not available for allowing the intraerythrocytic stages examination. However, as previously shown [67], description of new species without morphological identification is becoming a more and more common practice and has been also applied for other piroplasms [9,68], as morphological differences among the intraerythrocytic stages of piroplasms are mostly negligible. Moreover, low parasitemia in naturally infected wild hosts is unlikely to yield microscopically positive blood smears in the future.

The phylogenetic analysis of the mitochondrial genes showed that *B. pisicii* n. sp. is related to the so called “large” *Babesia* of dogs, forming a separate subclade in a sister position to *B. rossi*. The phylogeny based on the 18S rDNA sequences [42] showed that most feline-associated *Babesia* cluster in separate clades from *B. canis*, with the exception of *B. canis presentii*, *B. hongkongensis*, and *Babesia* sp. Western Cape. However, the 18S rDNA sequence analysis is able to distinguish between these three *Babesia* isolates and *B. canis*, which is not the case for *B. pisicii* n. sp.

Further studies are needed to clarify the host spectrum of *B. pisicii* n. sp., mainly its presence and clinical significance in domestic cats. Additionally, the elucidation of its life cycle and tick vector requires future attention. *Ixodes ricinus* and *I. hexagonus* were the only species found on wild felids in Romania [69] and both species are present in the area where *B. pisicii* n. sp. was found. Therefore, the vectorial competence of ticks in transmitting this new *Babesia* sp. remains to be investigated.

## 5. Conclusions

The current study indicates that European wild cats from Romania carry at least two *Babesia* species: *B. canis* and *B. pisicii* n. sp. Based on available genetic data, our recommendation is to avoid using the 18S rDNA detection for piroplasmid species differentiation and positive samples identified only based on this gene should be reported as *Babesia* sp. We encourage the implementation of molecular assays using mitochondrial genes, which, despite their lower sensitivity are able to clearly distinguish even between closely related piroplasm species.

## Figures and Tables

**Table 1:** Primer pairs, annealing temperatures, and expected length of amplicons.

Genetic Marker	Primers (Forward, Reverse)	Nucleotide Sequence (5′–3′)	Annealing Temperature	Product Size	Reference
18S rDNA	Bc_F1	CGTAGTTGTATTTTTGCGT	50 °C	≈430 bp	[33]
	GR2	CCAAAGACTTTGATTTCTCTC			
	Bc_F2	CATTTGGTTGGTTATTTCGTTTT	53 °C	376 bp	
	Bc_R1	GTTCCTGAAGGGGTCAAAAA			
18S rDNA	BT1F	GGTTGATCCTGCCAGTAGT	65 °C–55 °C	≈1730 bp	Modified after [8]
	BT outer R	GGAAACCTTGTTACGACTTCTC			
	Piro0F2	GCCAGTAGTCATATGCTTGTCTTA	65 °C–55 °C	≈1670 bp	
	BT inner R	TTCTCCTTCCTTTAAGTGATAAG			
Cytb	Bc_cytb_F1	TGGTCWTGGTATTCWGGAATG	50 °C	≈700 bp	[34]
	Bc_cytb_R1	AAGMYARTCTYCCTAAACATCC			
	Bc_cytb_F2	RATKAGYTAYTGGGGAGC	48 °C	≈580 bp	
	Bc_cytb_R2	GCTGGWATCATWGGTATAC			
COI	Bab_For1	ATWGGATTYTATATGAGTAT	45 °C	1250 bp	[34]
	Bab_Rev1	ATAATCWGGWATYCTCCTTGG			
	Bab_For2	TCTCTWCATGGWTTAATTATGATAT	49 °C	980 bp	
	Bab_Rev2	TAGCTCCAATTGAHARWACAAAGTG			

**Table 2:** Pairwise nucleotide sequence identities (%) of Cytb (lower left) and COI (upper right) genes for *B. canis*, *B. vogeli*, *B. pisicii* n. sp., and *B. rossi*.

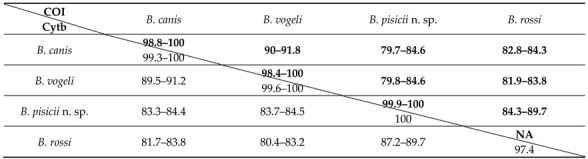

Pairwise nucleotide sequence identities of COI gene are set in bold.

## Data Availability

The datasets supporting the conclusions of this study are included in this published article (and its supplementary information files).

## Supplementary Materials

The following are available online at https://www.mdpi.com/article/10.3390/microorganisms9071474/s1, Figure S1: Sensitivity of the nested PCR assay targeting the 18S rDNA fragment of 376 bp. Table S1: Pairwise amino acid sequence identities (%) of Cytb (lower left) and COI (upper right) genes for *B. canis*, *B. vogeli*, *B. pisicii* n. sp. and *B. rossi*.
