# Supplementary material for: Babesia pisicii n. sp. and Babesia canis Infect European Wild Cats, Felis silvestris, in Romania
Source: Microorganisms. 2021 Jul 9;9(7):1474. doi: 10.3390/microorganisms9071474 (PMC8308005; doi:10.3390/microorganisms9071474)

Sensitivity of the nested PCR assay targeting the 18S rDNA fragment of 376 bp; L – 100 bp DNA ladder; B_{DNA} – background DNA isolated from *Babesia* spp. negative wild cats; C- – non-template negative control; 1, 10, 10^2, 10^3, 10^4, 10^5, represents the number of template DNA copies in each reaction.

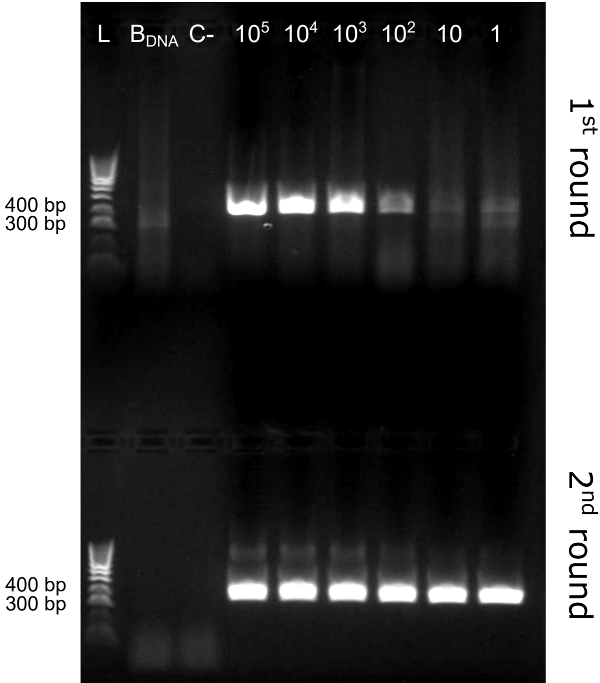

Supplement: Supplementary file 1 [file microorganisms-09-01474-s001.zip › Figure S1.pdf]
