# Supplementary material for: Babesia pisicii n. sp. and Babesia canis Infect European Wild Cats, Felis silvestris, in Romania
Source: Microorganisms. 2021 Jul 9;9(7):1474. doi: 10.3390/microorganisms9071474 (PMC8308005; doi:10.3390/microorganisms9071474)
Supplement: Supplementary file 1 [file microorganisms-09-01474-s001.zip › Table S2.pdf]

Table S2 (Supplementary file 2)

Pairwise amino acid sequence identities (%) of Cytb (lower left) and COI (upper right) genes for *B. canis*, *B. vogeli*, *B. pisicii* n. sp., and *B. rossi*

Cytb \ COI	***B. canis***	***B. vogeli***	***B. pisicii* n. sp.**	***B. rossi***
B. canis	98.3-100 / 99.5-100	96.6-98.3	87.4-93.4	92.5-94.1
B. vogeli	91.3-93.5	99.3-100 / 99.4-100	87.4-95.2	93.9-94.6
***B. pisicii* n. sp.**	87.1-89.4	89.2-91.4	99.7-100 / 100	91.3-97.9
B. rossi	86.3-89	83.3-87.1	91.7-94.1	NA / 95.8
